# Overall survival of recurrent/metastatic head & neck squamous cell carcinoma patients progressing after ≥ 1 line of systemic therapy, treated with MVX-ONCO-1, a novel, first in class cell encapsulation-based immunotherapy: results of SAKK 11/16, a phase IIa trial

**DOI:** 10.1186/s40164-025-00703-x

**Published:** 2025-08-31

**Authors:** Eugenio Fernandez, Rémi Vernet, Muriel Urwyler, Olivier Von Rohr, Emily Charrier, Marie-Claude Belkouch, Valentin Saingier, Fabien Courtout, Claudio DeVito, Virginie Ancrenaz, Nicolas Dulguerov, Wolfram Karenovics, Julien Grogg, Jessica Renaux, Katrin Gobat, Gisela Müller, Tomas Brezina, Tamara Rordorf, Markus Joerger, Olivier Michielin, Jean Villard, Nicolas Mach

**Affiliations:** 1 Division of Oncology, Geneva University Hospitals, Geneva, Switzerland; 2 Translational Research Centre in Onco-Hematology, University of Geneva, Geneva, Switzerland; 3 Swiss Cancer Center Léman (SCCL), Geneva and Lausanne, Switzerland; 4 Division of Clinical Pathology, Diagnostic Department, Geneva University Hospitals, Geneva, Switzerland; 5 Department of Otorhinolaryngology-Head and Neck Surgery, Geneva University Hospitals, Geneva, Switzerland; 6 Thoracic Surgery, Geneva University Hospitals, Geneva, Switzerland; 7 MaxiVAX SA, Geneva, Switzerland; 8 Competence Center Swiss Group for Clinical Cancer Research (SAKK), Bern, Switzerland; 9 Department of Medical Oncology and Hematology, University Hospital Zurich, Zurich, Switzerland; 10 Department of Medical Oncology & Hematology, Cantonal Hospital, St. Gallen, Switzerland; 11 Clinical Cell Therapy Lab, Geneva University Hospital, Geneva, Switzerland; 12 Division of Oncology, Geneva University Hospitals and Medical School, Rue Gabrielle Perret-Gentil 4, 1211 Geneva, Switzerland

**Keywords:** Immunotherapy, Cell encapsulation device, Granulocyte–macrophage colony stimulating factor, Autologous irradiated tumor cells, Advanced solid tumors

## Abstract

**Background:**

Over the past two decades, most cancer vaccines have failed to be developed clinically. The lack of efficient priming with specific tumor antigens and/or weak adjuvants may explain this poor success rate. MVX-ONCO-1, a personalized cell-based vaccine, combines inactivated autologous tumor cells and encapsulated allogeneic human cells genetically engineered to produce granulocyte–macrophage colony stimulating factor (GM-CSF). This unique technology allows sustained local delivery of strong adjuvant at the vaccination site. The combination of inactivated autologous tumor cells and potent local adjuvant delivery addresses these two unmet critical steps and may recapitulate in patients the successful combination observed in experimental models.

**Methods:**

The SAKK 11/16, a Phase IIa trial with Overall Survival (OS) as the primary endpoint was the first efficacy study evaluating MVX-ONCO-1. Patients with Recurrent/Metastatic Head and Neck Squamous Cell Carcinoma (R/M HNSCC) progressing after at least one line of systemic therapy were enrolled with 50% of patients alive at 26 weeks as the primary objective.

**Results:**

In this hard-to-treat population, SAKK 11/16 met the primary endpoint, with 68.8% of patients alive at 6 months. The median OS was 11.4 months, with 32% of the patients alive after 18 months. Complete and partial responses were observed on MVX-ONCO-1 monotherapy.

Moreover, all patients who developed a positive DTH reaction to their tumor cells upon vaccination survived at 12 months. Additionally, patients living for more than 12 months had higher circulating antibody titers against tumor-associated antigens. Explorative analysis looking at median OS from the start of anti-PD-1 therapy was 21.7 months.

In addition, no new safety signals with no systemic adverse events (AE) related to the treatment and no manufacturing issues were observed in this multicenter trial.

**Conclusions:**

These findings suggest that MVX-ONCO-1 can induce a coordinated immune response with clinical benefits as a standalone treatment, leading to prolonged survival. This effect may be enhanced by previous exposure to immune checkpoint inhibitors.

*Trial registration* (ClinicalTrials.gov): NCT02999646.

## Background

Therapeutic cancer vaccines have been viewed as an unrealistic goal over the last 40 years, with many failures despite tremendous progress in dissecting cancer immunology and biotechnology tools. The lack of strongly immunogenic cancer-specific targets, lack of efficient adjuvants, and the need for patient-specific, customized products are potential causes for either poor efficacy or challenges in moving beyond exploratory Phase I clinical trials [1–3]. The early success of mRNA-based, personalized cancer vaccines in the adjuvant setting, in combination with immune checkpoint inhibitors, has led to renewed interest in the field [4].

Here, we report the results of SAKK 11/16, the first efficacy trial of MVX-ONCO-1, a personalized cell-based cancer vaccine using cell-encapsulation technology, with OS at 26 weeks as the primary endpoint.

Over the past 20 years, efficient cancer vaccination has been observed in murine models across all tumor types via the use of genetically modified tumor cells. Indeed, irradiated tumor cells engineered to produce the cytokine GM-CSF [5, 6] induce long-lasting protective immunity with prolonged survival in murine tumors, including melanoma, sarcoma, leukemia, lymphoma, neuroblastoma, glioblastoma, and many carcinomas, such as those of the lung, ovary, stomach, liver, breast, colon, urothelial, and kidney. Such strategies rely on two critical factors: cancer-specific antigens provided by irradiated cancer cells, and sustained, controlled, local delivery of GM-CSF at the vaccination site for 5–7 days. While the use of cancer-specific antigens is well accepted in the scientific community, fine tuning of the adjuvant is often overlooked. Indeed, the potent adjuvant effect of GM-CSF is strongly correlated with the delivery method. Local production of low level of GM-CSF at the vaccine site has very potent immunostimulatory effects, triggering a coordinated cellular and humoral immune response, while excessive local GM-CSF and/or systemic GM-CSF exposure has a negative impact on immunostimulation, leading to tolerogenic effects [7, 8].

Many tumor vaccine trials in patients with advanced cancer have failed to recapitulate these two critical parameters. Indeed, many studies have used high-dose recombinant GM-CSF as an adjuvant for peptide-based vaccines, and several cell-based cancer vaccine trials have used unprotected allogeneic cancer cells engineered to produce GM-CSF (GVAX platform [9, 10]). In both strategies, neither the patient’s own tumor-specific antigens nor sustained, local, controlled GM-CSF production at the vaccination site over several days could be achieved, leading to weak immunostimulation and negative trials.

**Fig. 1:**
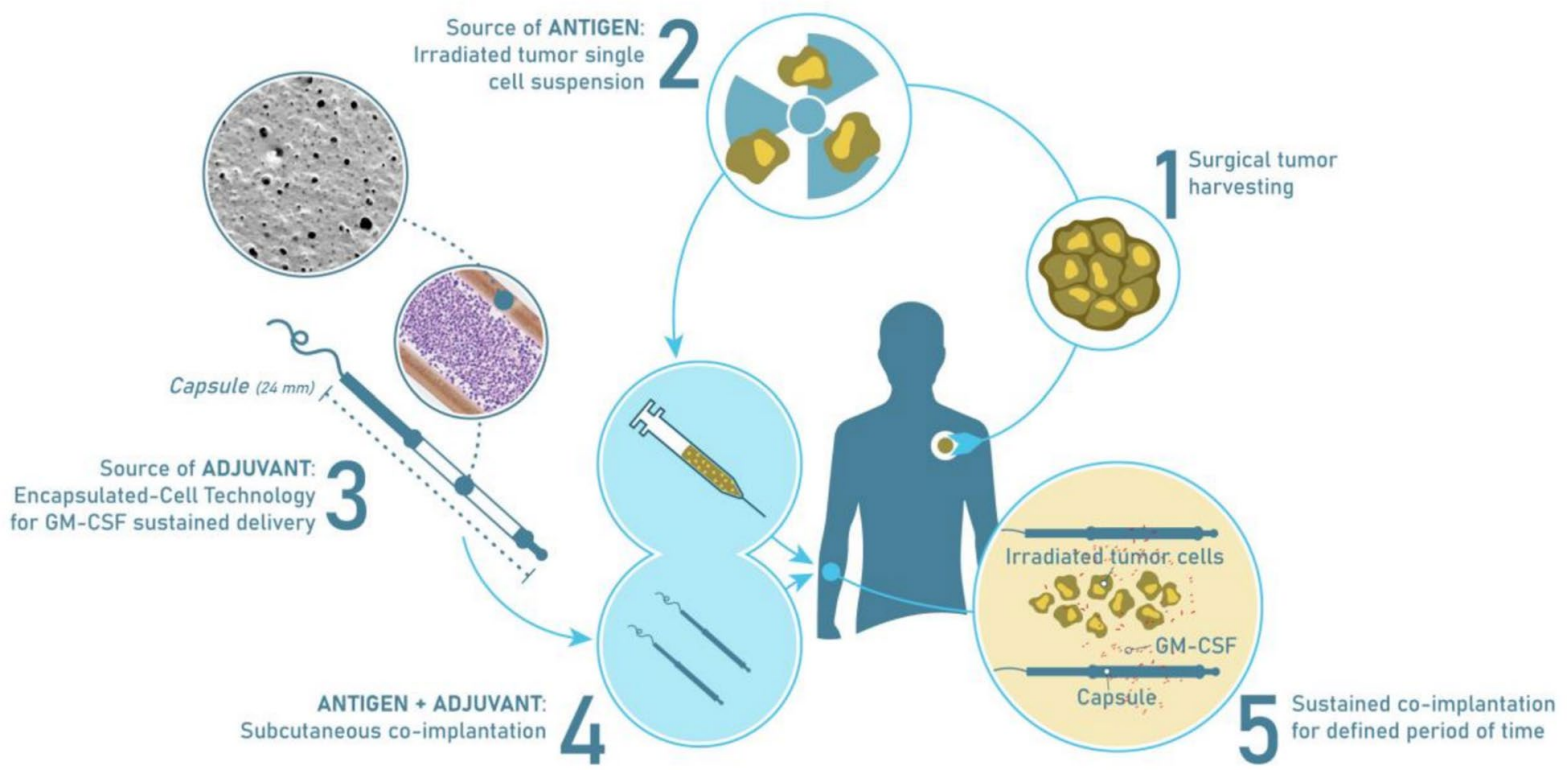
Graphic illustration of Investigational Medicinal Product (IMP)

MVX-ONCO-1 is an innovative vaccination technology that fulfills the following features (Fig. 1).

MVX-ONCO-1 focuses on leveraging the patient's own tumor-specific antigens, ensuring a highly personalized treatment approach tailored to the unique characteristics of individual tumors without the need to identify, isolate, or manufacture defined antigens. This treatment can be applied to any tumor type, is not restricted to Human Leucocyte Antigen (HLA), and can be applied in both adjuvant and metastatic settings. This personalized targeting is complemented by the controlled and sustained delivery of GM-CSF at the vaccination site over a period of 7 days without the systemic effects of the adjuvant. This sustained, controlled delivery was achieved by macrocapsules loaded with MVX-1, a human cell line engineered to produce GM-CSF. While antigens are personalized, capsules loaded with GM-CSF-secreting cells are similar in all patients and can be produced in large batches. Both components can be stored under frozen conditions. The vaccination is achieved by implanting the irradiated cells and the loaded capsules subcutaneously next to each other, under healthy skin, away from any tumor deposit, in a brief 30-min outpatient procedure.

Moreover, the manufacturing process of MVX-ONCO-1 is straightforward, eliminating the need for complex genetic engineering of patient tumor cells or prolonged culture. This streamlined approach not only facilitates production, but also ensures reproducibility. The MVX-ONCO-1 processing time was 7 days, which was set by biosafety analysis, enabling swift transition from tumor cell harvesting to treatment administration. This timeline enhances the feasibility of timely intervention and minimizes delays in patient care.

Data from the First in human Phase I study, which included 34 patients with various advanced refractory solid malignancies, were recently published [11, 12]. The primary endpoints of safety and feasibility were met. In addition, interesting clinical findings, including partial responses (PR), clear evidence of immune education, and prolonged survival were observed. Of particular interest, two out of two patients with R/M HNSCC, progressing after chemotherapy and/or anti-PD-1 therapy, exhibited intriguing clinical benefits. More specifically, one patient achieved PR, while another survived for more than 7 years without anticancer therapy for over 5 years.

The combination of interesting early clinical data, minimal toxicity, and signs of immune education prompted us to plan, design, and perform SAKK 11/16, a multicenter Phase IIa efficacy study with OS as the primary endpoint for patients with R/M HNSCC progressing after at least one line of systemic therapy [13].

## Methods

### Study design and participants

SAKK 11/16 is a multicenter, prospective, single arm, open label phase II trial in patients with R/M HNSCC progressing after first line therapy for metastatic disease (chemotherapy or immunotherapy). This patient’s population has a poor prognosis, with no well-defined standard treatment and a survival between 6 and 9 months.

### Sample size calculation

Originally, a single-arm, single-stage phase II design according to A’Hern [14] was used to test the following one-sided hypotheses:

- H0: The OS at 26 weeks ≤ 30%.
- H1: The OS at 26 weeks ≥ 50%.

Assuming an OS at 26 weeks of 50% under treatment with MVX-ONCO-1, 39 patients were required to test these hypotheses, with a one-sided type-I error (α) of 0.05 and 80% power.

In an unplanned interim data review, 10 patients were evaluated and 7 of them OS longer than 26 weeks. Based on these numbers, the sample size was re-estimated using the conditional power method described by Kieser et al. [15]. The new sample size obtained with the same power and the same type-I error as above and based on the interim result was a total of 20 patients.

An exact binomial test was used for analysis. The null hypothesis in the adaptive design could be rejected if, among the 20 patients, there were ≥ 11 survivors (patients with OS ≥ 26 weeks). Seven survivors were already present in the interim sample. At least four survivors of the additional 10 patients were required.

### Statistical methods

For the primary analysis, a one-sided exact binomial test was performed to test the null hypothesis that the OS at 26 weeks was ≤ 30%. The proportion of patients alive 26 weeks after registration was reported together with an exact 95% lower confidence bound.

For all time-to-event endpoints, the median value was estimated using the Kaplan–Meier (KM) method, along with a 95% confidence interval (CI). The number of events at each endpoint is presented descriptively by frequency and percentage.

The initial sample size was calculated using R 3.1.0 and the clinfun package version 1.0.6. The re-estimation was calculated using R 4.1.2. All analyses were performed using SAS 9.4 (SAS Institute, Cary, NC, USA) and R 4.3.3 (The R Foundation; www.r-project.org" www.r-project.org).

### Study population

Safety population: The safety population is defined as all patients who received at least one trial of treatment. Full Analysis Set (FAS): The FAS is defined as all registered patients who received at least one dose of trial treatment (modified intention-to-treat principle, mITT) excluding patients with major eligibility violations. At least one dose of trial treatment was defined as at least 1 day of implantation without capsule removal. Per Protocol Set (PPS): The per protocol set is based on the FAS, excluding the following patients: patients with major protocol violations, patients with incomplete trial treatment (defined as < 6 implantations of 2 capsules at each implantation, or at least one vaccination explantation < 6 days after implantation).

### Procedure

MVX-ONCO-1 treatment involves two key elements, as previously described [12] (Fig. 1). First, tumor cells are obtained from a readily accessible tumor site and processed into a single-cell suspension. These cells were lethally irradiated (100 Grays), aliquoted, and stored in liquid nitrogen prior to treatment. Second, genetically modified human K562 cell line that secrete GM-CSF were loaded into the biocompatible macrocapsules. The treatment regimen included two macrocapsules implanted subcutaneously 1 cm apart and irradiated autologous tumor cells injected between the capsules. Treatment was administered weekly for four weeks, with additional immunizations at weeks 6 and 8. The macrocapsules were removed one-week post-implantation. Delayed Type Hypersensitivity (DTH) with irradiated autologous tumor cells was performed 3 times, before treatment, and then at weeks 6 and 12.

### Endpoints and assessments

The primary endpoint was Overall Survival (OS) at 26 weeks, which was defined as the percentage of patients alive 26 weeks after registration. The study is positive if > 50% of patients were alive at this time point. The final analysis was performed after all patients underwent tumor assessment at 52 weeks after the start of treatment.

The secondary endpoints are Time to subsequent therapy, Duration of response according to Response Evaluation Criteria in Solid Tumors (RECIST) 1.1, Objective response rate (ORR) according to RECIST1.1 at 6, 13, 26, 39, and 52 weeks after registration, Disease control rate (DCR) according to RECIST 1.1 at 6, 13, 26, 39, and 52 weeks after registration, Best overall response according to RECIST 1.1, Objective response according to iRECIST (iOR), Progression-free survival (PFS) according to RECIST 1.1, Progression-free survival according to iRECIST (iPFS), PFS at 6, 13, 26, 39, and 52 weeks after registration according to RECIST 1.1, OS, PFS under the first subsequent treatment according to RECIST 1.1, Best overall response under the first subsequent treatment according to RECIST 1.1. Adverse and serious adverse events (SAE).

The exploratory endpoints were immune monitoring and DTH test at baseline (pre-treatment), week 6, and after the end of treatment. Site biopsy for biomarkers at baseline (pre-treatment) and after treatment (4 weeks after removal of the last implanted capsules), tumor histology at baseline, weeks 13, 26, and 52 after registration (optional, to be done only if no risk for the patient), QoL, Pain, and Tumor response according to RECIST 1.1 during follow-up, whenever such data are available, in relation to subsequent treatment status, when applicable.

Safety was evaluated by clinical assessments, vital signs, local and systemic tolerance, laboratory tests, and electrocardiograms conducted from baseline until week 18. Patients could discontinue the study treatment for unacceptable toxicity, pregnancy, or patient decisions. Adverse events were graded using the Common Terminology Criteria for Adverse Events (CTCAE) v.4.0 and reported using the Medical Dictionary for Regulatory Activities (MedDRA) v25.0, until 30 days after removal of the last implanted capsules, while related AEs and SAEs were recorded until the end of participation in the study. Patients were then followed up for survival status and SAE until death or year 5, whichever occurred first.

Efficacy was assessed by monitoring patient survival at 6, 12, and 18 months and disease status using Response Evaluation Criteria in Solid Tumors (RECIST) 1.1 at baseline and then at weeks 6, 12, and 18, with a cut-off date of June 29th, 2023. Additional information on the tumor status beyond week 18 was obtained for specific subjects after the implementation of a dedicated amendment.

### Delayed type hypersensitivity

For DTH testing, 1 × 10^6^ irradiated autologous tumor cells were injected intradermally into healthy skin before (baseline), during (week 6), and after end of treatment. Positivity (≥ 5 mm diameter) was assessed by measuring erythema, induration, and ulceration at 48–72 h, following international guidelines, with punch biopsies taken to analyze immune cell recruitment.

### Multiplex immunofluorescence staining and acquisition

Multi-IF tissue staining was performed using the BOND RX stainer (Leica Biosystems) in a fully automated fashion and involved sequential steps of staining with each primary antibody using TSA-OPAL dyes from Akoya Biosciences. FFPE tumor sections (4 μM) were deparaffinized, rehydrated, and antigen retrieval was performed using ER1/ER2 buffer. A blocking step was performed followed by primary antibody incubation. A secondary antibody, coupled to a Horseradish Peroxydase, was applied, followed by tyramide signal amplification and OPAL fluorophores. This sequence was repeated for the six primary antibodies. A tonsil slide was used as a positive control for each run. The sections were counterstained with spectral DAPI (Akoya Biosciences). Whole slides were imaged at 20 × magnification using a Vectra Polaris multispectral scanner (Akoya Biosciences).

### Ex-vivo modified IFN-ɣ enzyme-linked immuno spot (ELISpot)

Peripheral blood mononuclear cells (PBMCs) were harvested, frozen, and then thawed in X-Vivo 15 (Lonza, BE02-060F) before overnight resting (5% CO₂, 37 °C). PBMCs were co-incubated with freshly thawed autologous irradiated tumor cells (1:2 ratio) for 24 h in pre-coated 96-well plates (Mabtech). After washing according to the manufacturer’s instructions, the plates were analyzed using a Mabtech IRIS 2 reader. Spot-forming units (SFU) were calculated after subtracting the background from the negative controls (PBMCs or tumor cells only).

### Auto-antibody analysis—seromic profiling

Antibody profiling of autoantibodies was outsourced at Cambridge Protein Arrays and was performed on HuProt™ v4.0 Human Proteome Microarrays.

## Results

### Feasibility

The investigational personalized cancer vaccine evaluated in this trial is described in Fig. 1. Each vaccine was composed of 4 × 10^6^ irradiated autologous tumor cells, harvested from a metastatic lymph node or distant metastasis, and two biocompatible macrocapsules containing each 0.8 × 10^6^ cells, genetically engineered to secrete at least 20 ng of GM-CSF per day. Both cellular products were implanted next to each other, subcutaneously, away from any tumor deposit. Based on pre-clinical pharmacokinetics results, the capsules were removed after 7 days by pulling the string attached to it. The vaccination was repeated 6 times over 8 weeks (Week 1, 2, 3, 4, 6, and 8). The sites of implantation are in healthy skin, away from any tumor deposit. In order to stimulate several immunization areas, vaccinations are performed on the arms and thighs in a sequential manner: vaccine 1 on the left arm, vaccine 2 on the left thigh, vaccine 3 on the right thigh, and vaccine 4 on the right arm, etc. The patient did not receive maintenance therapy.

**Fig. 2:**
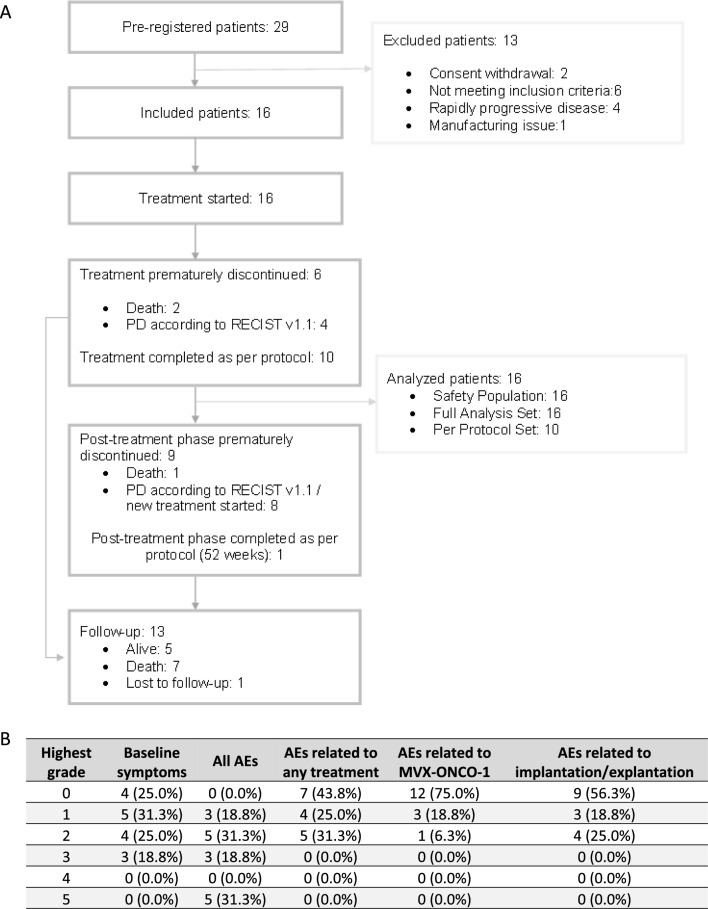
Study Patients: **A** CONSORT Diagram. **B** Baseline symptoms and AEs highest grade per patient—Safety population

Between 13/08/2018 and 25/07/2022, 16 patients were enrolled in the trial from 4 sites in Switzerland. The median follow-up time for the full analysis set (FAS) based on the reverse KM method was 23.8 months (95% CI 11.1-Not reached). The median observation time and range of censored patients in the FAS corresponds to 19.2 (11.1, 55.5) months. The patients’ medical history and baseline characteristics are summarized in Table 1, and the CONSORT diagram is shown in Fig. 2A.

**Table 1:** Patients’ medical history and baseline characteristics

Characteristic	Category	n	% or range
Sex	Female	1	6.3
	Male	15	93.8
Age	Median	59.5	42.0–76.0
WHO performance status	0	8	50.0
	1	6	37.5
	2	2	12.5
CPS score	< 1	3	18.8
	1–19	10	62.5
	≥ 20	2	12.5
	Missing	1	6.3
Location of primary tumor	Hypopharynx	2	12.5
	Oral cavity	7	43.8
	Oropharynx	7	43.8
HPV result for Oropharynx Location	Negative	2	28.6
	Positive	3	42.8
	Unknown	2	28.6
Did a relapse occur?	No	10	62.5
	Yes	6	37.5
Metastatic disease?	No	2	12.5
	Yes	14	87.5
Location(s) of metastasis (more than one applicable)	Bone	3	18.8
	Distant lymph node	7	43.8
	Kidney	1	6.3
	Liver	2	12.5
	Lung	11	68.8
	Pleura	0	0.0
	Soft tissue	1	6.3
P 16 result	Negative	8	50.0
	Positive	5	31.3
	Unknown	3	18.8
Time from diagnosis to registration (years)	Median	2.1	0.8–9.0
Previous systemic therapy	Yes	16	100.0
Pre-treatment with check-point inhibitor	No	2	12.5
	Yes	14	87.5
Previous radiotherapy	No	2	12.5
	Yes	14	87.5
Previous tumor surgery	No	8	50.0
	Yes	8	50.0
Relevant concomitant diseases	No	4	25.0
	Yes	12	75.0

As required by the protocol, all patients had metastatic disease with at least one tumor lesion amenable to minimally invasive, aseptic, surgical resection and at least another measurable metastasis according to the Response Evaluation Criteria in Solid Tumors (RECIST), RECIST1.1, and iRECIST criteria.

The trial showed that personalized cell-based cancer immunotherapy using encapsulated cell technology is feasible in a multicenter setting. Indeed, the SAKK 11/16 study team was able to successfully coordinate the manufacturing and shipping of therapeutic biological material from and to the sites.

### Safety

No new safety signals were observed in this trial compared with the data reported in patients treated in the Phase I study [12]. No systemic AEs related to the investigational medicinal product (IMP) were reported (Fig. 2B). Systemic AEs were linked to either underlying cancer or other medical conditions. Some patients experienced local irritation, and for two patients, harvesting the capsules on day 8 was challenging, and required ultrasound guidance.

This trial showed that this novel cancer immunization strategy is safe in this population with R/M HNSCC. With now 50 cancer patients treated with MVX-ONCO-1 (34 in Phase I and 16 in Phase IIa), we obtained a good overview of the safety parameters, with no acute or late systemic adverse effects related to the therapeutic procedures.

Quality of Life was reported via standardized questionnaires, as well as pain at implantation and explantation (data not shown).

### Efficacy

The primary analysis was performed for the FAS population. All patients were follow-up at least 11 months, with only one patient lost to follow-up after 413 days.

Most patients were exposed to more than two lines of therapy for metastatic disease, with 87.5% enrolled after progression on anti-PD-1 therapy.

**Fig. 3:**
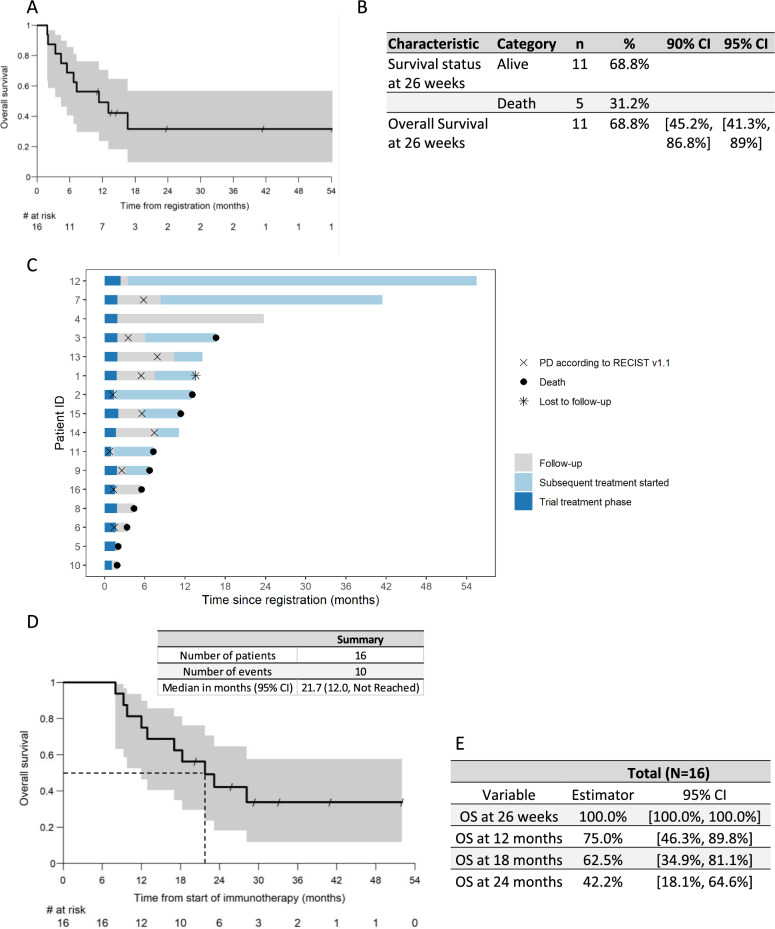
Efficacy results—Primary Endpoints: **A** Kaplan–Meier plot for Overall Survival. **B** Descriptive summary of Overall Survival at 26 weeks. **C** Swimmer plot. Explorative analysis **D** Median Overall Survival and Kaplan–Meier plot for Overall Survival from start of Immunotherapy. **E** Overall Survival from start of Immunotherapy at fixed time point

In this hard-to-treat population, the trial achieved the primary endpoint of OS at 26 weeks. This positive milestone was achieved ahead of schedule after only 16 patients were enrolled, 11 out of 16 patients reached the 26 weeks OS timeline, leading to an early termination of enrollment as the primary endpoint was met (OS at 26 weeks 68.8%) (Fig. 3A). As the lower boundary of the two-sided 90% CI is above 30%, the null hypothesis that the OS at 26 weeks is ≤ 30% can be rejected. Furthermore, the two-sided 95% CI was above 30% (Fig. 3B).

The impact of this novel personalized anticancer vaccination on tumor shrinkage is unknown. Indeed, it has been well established that the response rate or progression free survival (PFS) may not be reliable surrogate markers for OS in the era of immunotherapy [16]. In this study, we observed few objective responses, with one (6.3%) complete response (CR) and one (6.3%) partial response, with 10 patients (62.5%) having stable disease (SD) as the best clinical response and 4 patients (25%) having tumor progression as the best response to treatment. The disease control rate was defined as the proportion of patients who achieved CR, PR, or SD at the respective time points after registration and before starting a new anticancer treatment. The DCR at 26 weeks was 3 (18.8%), with a 95% CI between [4–45.6%].

The median OS in this trial was 11.4 months at the data cut-off (with 5 patients alive and one lost to follow-up) (Fig. 3C). Patient 4 had multiple lung and mediastinal lymph node metastases, with a clear progression after nivolumab therapy, achieved complete remission, lasting for 16.5 months and was ongoing at the time of data cut-off.

We observed unusual clinical courses, some patients experienced tumor stabilization and a tumor response upon subsequent palliative immunotherapy or chemotherapy. Indeed, in this heavily pretreated population, palliative anticancer therapy leads to tumor control or partial response in several patients. In this setting, patient 12, showing SD as the best response upon treatment with MVX-ONCO-1, subsequently received nivolumab despite negative PD-L1 expression on tumor cells and the tumor microenvironment (CPS = 0). This patient experienced an unusual subsequent complete response, with the disappearance of large bilateral pulmonary metastases. This complete remission was long-lasting (> 3 years and ongoing at the data cut-off) (Fig. 3C).

As 14 out of 16 patients were enrolled with progressive disease (PD) after anti-PD-1 therapy as a previous line of therapy for metastatic disease, we also analyzed survival from the beginning of immune-checkpoint inhibitor therapy. This exploratory analysis calculated OS at fixed time points from the beginning of anti-PD-1 therapy and revealed a median OS of 21.7 months, much longer than those previously reported in the literature (Fig. 3D, E, and Discussion).

Additional observations were made for patients who received palliative subsequent therapy to evaluate the median time to the next treatment (chemotherapy, cetuximab, nivolumab, or other clinical studies). From registration until the documented start of subsequent therapy, the median time was 7.5 months, with a 95% CI ranging from 3.5 to 8.3 months.

### Exploratory endpoints

#### Immunomonitoring

All the secondary endpoints were analyzed based on the FAS if not stated otherwise.

To characterize long survivors, defined as patients alive for more than 12 months, versus short survivors, defined as patients living less than 12 months, extensive translational analyses were performed. Among the various tests performed, we examined immune parameters such as DTH, *ex-vivo* lymphocyte activation (ELISpot), and the humoral response.

**Fig. 4:**
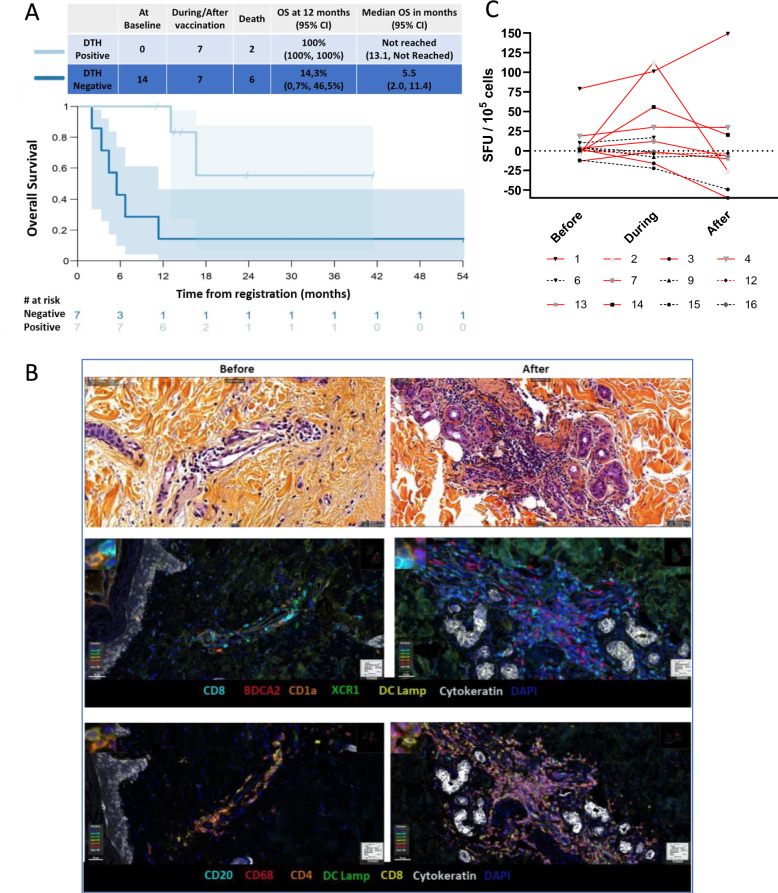
*In-vivo & *In vitro stimulation of Patient Immune cells. **A** Kaplan–Meier plot of Overall Survival by DTH category (Negative and Positive upon vaccination). Overall Survival at 12 months and Median Overall Survival by DTH category. **B** Representative Multiplex immunofluorescence pictures of immune cells infiltrate in the punch skin biopsy at the site of DTH before and after treatment. The top two images represent hematoxylin phloxine saffron staining, the middle and bottom images are both multiplex immunofluorescence staining. **C** Quantification of PBMC IFN-γ secretion by *ex-vivo* modified ELISpot after stimulation with irradiated autologous tumor cells. Red lines indicate patients with survival higher than 12 months, while black lines indicate patients with survival less than 12 months. Full lines represent patients with a positive delayed-type hypersensitivity (DTH) response, and dotted lines represent those with a negative DTH response

The ability to mount an immune response towards autologous tumor cells, as measured by the DTH test may be predictive of prolonged survival. All included patients (16/16, 100%) had a negative DTH test result prior to treatment. Among 14 patients evaluable for DTH tests, 7 out of 8 patients (87.5%) who were alive at 12 months, developed a positive DTH reaction. Whereas, in DTH negative patients, 6 out of 7 (85.7%) died at earlier time points. No imbalances in clinical or biological parameters were observed between the patients who developed positive DTH and those who did not. Moreover, three DTH negative patients had one measurement instead of two after baseline due to rapid progression or early death. The median OS for DTH negative patients was 5.5 months (95% CI 2.0–11.4 months) while the median OS was not reached for DTH positive patients (95% CI 13.1-NR) (Fig. 4A).

Skin punch biopsy of the DTH site was performed and characterized in some patients. Representative images are shown in Fig. 4B. This analysis is not quantitative but illustrates the influx of immune cells, such as CD4 + and CD8 + T cells, B cells and Antigen Presenting Cells when the DTH became positive. These findings confirm that positive DTH correlates with the recruitment of immune cells, which is a key factor for immune education towards tumor cells.

*Ex-vivo* ELISpot analysis confirmed that immune activation was effectively initiated by the treatment. As illustrated in Fig. 4C, 67% of patients who survived for more than 12 months presented an increase in T-cell reactivity from baseline (before treatment) to other time points during treatment, compared to non-survivors who showed no changes.

Interestingly, for patients who survived more than 12 months and developed DTH positivity, 5 out of 7 patients (71.4%) developed ELISpot positivity. Conversely, among patients who did not survive more than 12 months, 0 out of 6 patients (0%) showed DTH positivity, and only 1 out of 6 patients (16.7%) developed ELISpot positivity. Additionally, 5 out of 5 patients (100%) who developed both DTH, and ELISpot positivity survived for more than 12 months, whereas 3 out of 4 patients (75%) who lacked both tests did not survive beyond 12 months. These findings suggest a potential link between immune responses and prolonged survival.

**Fig. 5:**
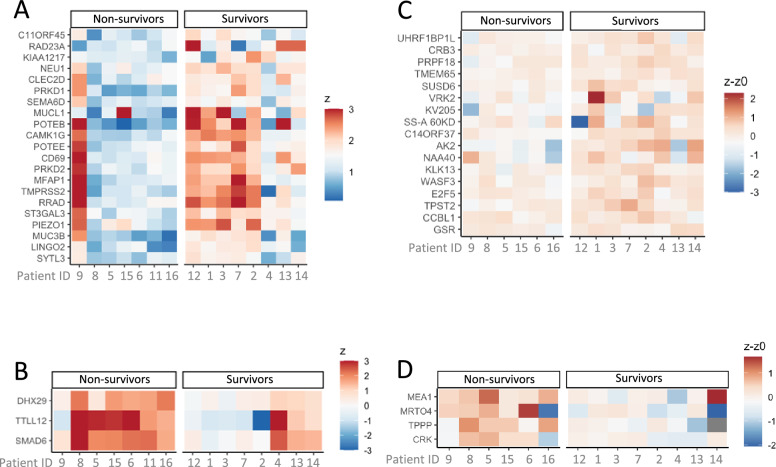
Antibodies Profile Level at Baseline **A** Higher in survivors (survival higher than 12 months) compare to non-survivors (survival lower than 12 months). **B** Higher in non-survivors compare to survivors. Antibodies Profile Top changes during treatment. Baseline timepoint compare to during treatment timepoint (Patient 11 has only baseline seromic sample, no comparison was done). Increase of antibodies quantity **C** in survivors compare to non-survivors and **D** in non-survivors compare to survivors

Finally, there are scientific evidences that GM-CSF cell-based cancer immunotherapy can also stimulate an antibody-based immune response [17–20]. Therefore, as an explorative analysis, we investigated the antibody immune response using quantitative seromic technology. This analysis revealed that most long-term survivors, over 12 months, had higher antibody titers (Fig. 5A) against several tumor associated antigens, such as mucin proteins MUCL-1 [21], MUC3B [22] and the cancer testis antigen POTEE [23] at baseline, than patients who survived for less than 1 year. In addition, long-term survivors also have antibodies against proteins known to promote cancer growth, such as PIEZO1 [24], aggressiveness such as TMPRSS2 [25], and Epithelial Mesenchymal Transition such as KIAA1217 [26] or C11orf45 [27], which are biomarkers related to immune checkpoint proteins. Moreover, patients who survived for less than 1 year had higher antibody titers for SMAD6 [28] (Fig. 5B), suggesting an increased immune reaction against the SMAD6 protein in these patients. This protein is directly associated with poor patient prognosis.

Interestingly, a high antibody titer against genes involved in immune modulation, such as SEMA6D^22^ as well as an increase in the antibody titer during treatment (Fig. 5C, D) against the transmembrane SUSD6 protein was also observed in survivors over 12 months compared to non-survivors. As described in the literature [29, 30], SUSD6 expression is linked to a decrease in MHC-I expression and blockage of SUSD6 activity induces a stronger T cell response and synergizes with anti-PD-1 therapy.

## Discussion

The survival of R/M HNSCC patients refractory to first-line chemo/immunotherapy is poor [31]. Data from prospective clinical trials in second-line R/M HNSCC have dismal survival, with median OS ranging from 7.6 months for durvalumab [32] (Eagle), 7.7 months for nivolumab [33, 34] (Checkmate141), 8.0 months for avelumab [35, 36] (Javelin) to 8.4 months for pembrolizumab [37, 38] (Keynote 040) with 2 years survival rate of 18.4%, 16,9%, 17.1%, and 17%, respectively. Cetuximab monotherapy has been approved in the US after platinum failure based on prospective single-arm studies with a median OS ranging from 5.2 to 6.1 months [39]. Palliative chemotherapy with methotrexate or taxane is often administered without documented benefits in randomized trials (European Society of Medical Oncology (ESMO) guidelines [40]). A randomized Phase 3 trial comparing Docetaxel ± Gefinitinib was negative, with a median OS of 6 months [41]. Therefore, there is a clear medical need for patients who fail to respond to chemotherapy and/or immunotherapy [42, 43]. Based on interesting clinical data observed in advanced refractory HNSCC patients treated with personalized, cell-based vaccination MVX-ONCO-1 in a Phase I study [35], we performed SAKK 11/16, a multicenter Phase IIA trial.

SAKK 11/16 achieved the OS primary endpoint ahead of the schedule. Indeed, in this hard-to-treat patient population with R/M HNSCC, progressing after at least one line of systemic therapy and 85% progressing after anti-PD-1 therapy, 68.8% (> 50%) were alive at 6 months. As the primary endpoint was reached after treating 16 patients, a decision to stop enrollment was made by the sponsor, and all treated patients followed the protocol. The median OS was 11.4 months (95% CI 4.4-NR) with some very long survivors as median 18 months OS rate was 31.6%. In addition, we observed complete and partial responses to the MVX-ONCO-1 vaccine monotherapy in this refractory patient population. As all patients received anti-PD-1 therapy (14/16 before entering the study and two in subsequent lines), we also performed an exploratory analysis to evaluate survival from the start of anti-PD-1 therapy to death or data cut-off. This analysis allowed for an indirect comparison with data from Keynote 40 [38] and Checkmate 141 [34], which evaluate pembrolizumab and nivolumab, respectively, in the second-line setting. A median OS of 21.7 months from the start of anti-PD-1 therapy for patients enrolled in SAKK 11/16 was observed, a striking difference from the 7–8 months median OS reported in randomized Phase III studies with anti-PD-1 therapy as the second-line treatment.

In addition, no new safety signals, and no systemic adverse events related to the study treatment were observed. No manufacturing or logistic issues were observed in this multicenter trial, with all treatments ready 10 days after the autologous tumor cells were harvested.

Analyzing the biological effects of MVX-ONCO-1 is critical to better understand clinical observations and prolonged survival. Currently, no prognostic/predictive factors have been identified in patients with MHNSCC who fail chemotherapy and/or immunotherapy.

Interestingly, we observed that all patients who mounted a cancer-specific immune response towards their own irradiated tumor cells, as measured by positive DTH upon treatment, were alive at 12 months. In sharp contrast, among patients with less than 12 months of survival, only 1 had a positive DTH. This translates into a major difference in the median OS between negative DTH (6 months) and positive DTH patients (median OS not reached, 95% CI 11.1-NR). Therefore, positive DTH after MVX-ONCO-1 therapy may be a strong predictive biomarker of prolonged survival. This benefit was observed in patients with both Human Papilloma Virus (HPV) positive and negative tumors.

In addition, we observed partial and complete responses upon vaccination and responses to subsequent anti-PD-1 therapy in PDL-1 negative tumors. These data raised the hypothesis that MVX-ONCO-1 may have the ability to trigger a clinically significant immune response as a monotherapy, and that this effect may be amplified in combination with immune checkpoint immunotherapies. Exploratory immunomonitoring, including seromic analysis, may provide an understanding of the underlying immune mechanisms involved. Such exploratory findings are hypothesis-generating to better characterize the immune stimulation and clinical activity observed in patients with very advanced disease. Local delivery of GM-CSF at the vaccination site by cell encapsulation technology may not only trigger a better recruitment and activation of Antigen Presenting Cells [44] but also help maintain a better MHC-I capacity, via downregulation of SUSD6.

SAKK 11/16 was the first personalized cell-based cancer vaccination trial that showed positive OS data in patients with relapsed-refractory metastatic Head & Neck squamous cell carcinoma, with a very good safety profile and signs of immune education.

Additional clinical studies in RM/HNSCC but also in other tumor types, and in combination with immune checkpoint inhibitors are required to better evaluate the potential benefits of this unique immunization strategy.

## Conclusion

This project underscores the significance of MVX-ONCO-1, a novel immunization strategy. The use of encapsulated, genetically modified allogeneic cells addresses the weaknesses of previous cancer vaccines. Combined with inactivated autologous tumor cells, it triggers a specific and clinically intriguing immune activation. This report may pave the way for a novel cancer immunotherapy potentially applicable to any cancer type.

In conclusion, MVX-ONCO-1 demonstrated notable safety, feasibility, and clinical benefits for patients with recurrent or metastatic cancer following the failure of standard anticancer treatment.

## Data Availability

No datasets were generated or analysed during the current study.

## Abbreviations

AE: Adverse event
CR: Complete response
CI: Confidence interval
CPS: Combined positive score
CTCAE: Common terminology criteria for adverse events
DCR: Disease control rate
DTH: Delayed type hypersensitivity
ELISpot: Enzyme-linked immuno spot
ESMO: European Society of Medical Oncology
FAS: Full analysis set
GM-CSF: Granulocyte–macrophage colony stimulating factor
HPV: Human Papilloma virus
HUG: Hôpital universitaire de Genève
IMP: Investigational medicinal product
iOR: Objective response according to iRECIST
iPFS: Progression-free survival according to iRECIST
KM: Kaplan–Meier
MedDRA: Medical dictionary for regulatory activities
ORR: Objective response rate
OS: Overall survival
PBMC: Peripheral blood mononuclear cells
PFS: Progression free survival
PPS: Per protocol set
PR: Partial response
RECIST: Response Evaluation Criteria in Solid Tumors
R/M HNSCC: Recurrent/Metastatic Head and Neck Squamous Cell Carcinoma
SAE: Serious adverse event
SAKK: Schweizerische Arbeitsgemeinschaft für Klinische Krebsforschung
SD: Stable disease
SFU: Spot-forming units
